# Diffusional Kurtosis Imaging in Idiopathic Normal Pressure Hydrocephalus: Correlation with Severity of Cognitive Impairment

**DOI:** 10.2463/mrms.mp.2015-0093

**Published:** 2016-02-03

**Authors:** Kouhei KAMIYA, Koji KAMAGATA, Masakazu MIYAJIMA, Madoka NAKAJIMA, Masaaki HORI, Kohei TSURUTA, Harushi MORI, Akira KUNIMATSU, Hajime ARAI, Shigeki AOKI, Kuni OHTOMO

**Affiliations:** 1 Department of Radiology, Graduate School of Medicine, University of Tokyo 7-3-1 Hongo, Bunkyo-ku, Tokyo 113-8655, Japan; 2 Department of Radiology, Juntendo University Graduate School of Medicine; 3 Department of Neurosurgery, Juntendo University Graduate School of Medicine; 4 Department of Radiological Sciences, Graduate School of Human Health Sciences

**Keywords:** normal pressure hydrocephalus, dementia, diffusional kurtosis imaging, diffusion tensor imaging

## Abstract

**Purpose:**

Diffusional kurtosis imaging (DKI) is an emerging technique that describes diffusion of water molecules in terms of deviation from Gaussian distribution. This study investigated correlations between DKI metrics and cognitive function in patients with idiopathic normal pressure hydrocephalus (iNPH).

**Materials and Methods:**

DKI was performed in 29 iNPH patients and 14 age-matched controls. Mini-mental state examination (MMSE), frontal assessment battery (FAB), and trail making test A (TMT-A) were used as cognitive measures. Tract-based spatial statistics (TBSS) analyses were performed to investigate the between-group differences and correlations with the cognitive measures of the diffusion metrics, including mean kurtosis (MK), fractional anisotropy (FA), apparent diffusion coefficient (ADC), axial diffusivity (AD), and radial diffusivity (RD).

**Results:**

In iNPH patients, FA and MK identified positive correlations with cognitive function in similar regions, predominantly in the frontal lobes (*P* < 0.05, corrected for multiple comparisons). The frontoparietal subcortical white matter showed significant correlations with FAB and TMT-A across more extensive areas in MK analyses than in FA. ADC, AD, and RD analyses showed no significant correlations with MMSE and FAB, while negative correlation with TMT-A was observed in the limited portion of the frontal deep white matter.

**Conclusion:**

Both FA and MK correlated well with cognitive impairment in iNPH. The observed differences between FA and MK results suggest DKI may play a complementary role to conventional FA and ADC analyses, especially for evaluation of the subcortical white matter.

## Introduction

Idiopathic normal pressure hydrocephalus (iNPH) is a clinical entity of unknown cause that is characterized by the triad of gait disturbance, cognitive impairment, and urinary incontinence.^1,2^ It is also associated with ventricular enlargement, flattening of high-convexity sulci, and periventricular hyperintensity on T_2_-weighted image (T_2_WI) in the absence of elevated cerebrospinal fluid (CSF) pressure.^3^ Although the gait disturbance usually precedes the other clinical signs and develops as the most prominent symptom,^4^ cognitive decline can also become a dominant symptom in some patients.^5^ Cognitive impairment in iNPH is considered as treatable by placement of a CSF shunt at least in the selected patients, though the degree of improvement after shunt surgery varies widely in different series.^6–11^ However, the pathological mechanism of such reversible cognitive impairment remains largely unknown.

Diffusion tensor imaging (DTI) has been applied to neurological and psychological diseases and is useful for detecting brain abnormalities that cannot be recognized by conventional T_1_ weighted image (T_1_WI) or T_2_WI. Previous DTI studies revealed increases of fractional anisotropy (FA) values in the corticospinal tract (CST) in patients with iNPH,^12–17^ which tended to return to normal after ventriculo-peritoneal shunt surgery.^12,13^ The increases in FA and axial diffusivity have been suggested to result from ventricular enlargement that mechanically compresses the tract and yields more directional water diffusion along it.^18^ Although several prior studies have reported the usefulness of diffusion magnetic resonance imaging (MRI) for evaluation of gait disturbance in iNPH,^12–17^ few studies have shown associations between diffusion abnormalities and cognitive impairments.^19^

Diffusion kurtosis imaging (DKI) is an emerging diffusion MRI technique. Unlike conventional DTI, which assumes that water diffusion has a Gaussian displacement probability distribution, DKI measures the deviation of the diffusion displacement profile from a Gaussian distribution. DKI provides unique parameters that describe the degree to which the water diffusion is non-Gaussian. This information is most commonly represented by the mean kurtosis (MK),^20–22^ which is related to properties of the tissue microstructure, for example, tissue complexity, the axonal water fraction, and the tortuosity of the extracellular space.^23,24^ Recent reports have suggested that DKI is sensitive to alterations of tissue microstructure in various kinds of diseases,^25–29^ especially in the areas of crossing fibers where conventional FA analyses have some difficulties intrinsic to the nature of FA as an index of directionality.^29^

The purpose of this study was to investigate tissue microstructural changes in patients with iNPH by using DKI, and to assess correlations between the diffusion metrics and cognitive scores.

## Materials and Methods

### Patients

The institutional review board approved this study and written informed consent was obtained from all participants. Twenty-nine patients with iNPH (13 males and 16 females; 76.4 ± 4.9 years old) and 14 age-matched control subjects (4 males and 10 females; 74.3 ± 3.5 years old) were recruited. Diagnosis of iNPH was made according to the criteria of probable iNPH provided by the Japanese Clinical Guidelines for Idiopathic Normal Pressure Hydrocephalus.^30^ Those who had a history of neurological disease other than iNPH or any significant findings (as observed on routine MR images) that might affect the brain were excluded. For all patients, cognitive measures (the mini-mental state examination [MMSE], frontal assessment battery [FAB], and trail making test A [TMT-A]) were recorded within 12 ± 17 days of the MR exam, before a CSF tap test was performed. In patients with iNPH, the results of cognitive measures were as follows (mean ± standard deviation): MMSE, 22.9 ± 4.5 (range 13–30); FAB, 12 ± 2.9 (range 7–17); TMT-A, 107 ± 66.5 s (range 30–320 s). Among the 29 patients, 24 showed relief of symptom after the tap test. Sixteen patients underwent shunt surgery, and 15 of them experienced significant clinical improvement (therefore, fulfilled the criteria for definite iNPH^30^).

Normal control subjects were required to be >60 years of age and have no neurological or psychological symptoms, history of neurologic diseases, or apparent abnormalities observed on conventional MR images.

### Data acquisition and post-processing

All patients underwent MR imaging with a 3-T unit (Achieva, Philips Medical Systems, Best, the Netherlands), equipped with an 8-channel head coil. DKI was acquired by using a single-shot, spin echo EPI sequence with five diffusion weightings (b = 500, 1000, 1500, 2000, and 2500 s/mm^2^) along 32 noncolinear directions, and 1 b = 0 s/mm^2^ volume (repetition time = 3000 ms, echo time = 80 ms, 20 axial slices, matrix 128 × 128, voxel size = 2 mm × 2 mm × 5 mm, parallel imaging factor 2, half-Fourier factor 0.667, number of excitation 2, acquisition time 824 s). The gradient length (δ) and the time between the two leading edges of the diffusion gradient (Δ) were held constant (Δ/δ = 39/28 ms). The signal-to-noise ratio (SNR) of the white matter was 35.0 ± 12.9, as measured by placing circular region of interest (ROI) of 10–20 voxel size in the centrum semiovale. Fast spin echo T_2_WI, spin echo T_1_WI, fluid-attenuated inversion recovery, and three-dimensional T_1_WI were obtained simultaneously and checked for the presence of other neurological disorders or lesions that might disturb diffusion MRI analyses.

**Fig. 1.**
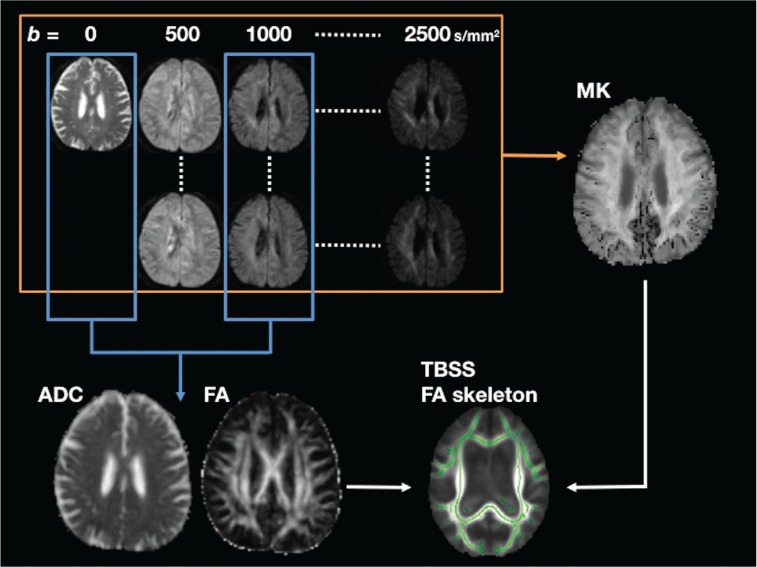
A flowchart of diffusion tensor imaging (DTI)/diffusional kurtosis imaging (DKI) image processing. The apparent diffusion coefficient (ADC) and fractional anisotropy (FA) maps were calculated by using b = 0 and b = 1000 s/mm^2^ data, while the entire data set was used for calculation of mean kurtosis (MK) maps. Following tract-based spatial statistics (TBSS) analyses of the FA maps, ADC and MK maps were also analyzed by applying the same transform as that for the FA maps (non-FA analyses function of TBSS).

DKI analyses were performed on an offline Windows PC, using dTV.II.FZR software (Medical Imaging Lab. HCU, Hiroshima, Japan).^31^ Prior to DKI calculation, the amount of noise (η) was estimated from the mean value of signals at air region in the image.^20^ The DKI parameters for a single direction can be determined with the following Equation (1) as described in previous studies^20,21^:

(1) $$S=\sqrt{{\left\{S_{0}\text{exp}\left(-bD_{\mathrm{app}}+\frac{1}{6}b^{2}{D_{\mathrm{app}}}^{2}K_{\mathrm{app}}\right)\right\}}^{2}+\eta^{2}},$$

where *S* is the diffusion-weighted signal intensity at the given *b* value, *S_0_* is the signal intensity at b = 0 s/mm^2^, *D_app_* is the mean diffusivity (MD), and *K_app_* is the apparent kurtosis coefficient (dimensionless). FA and apparent diffusion coefficient (ADC) values were calculated separately using only data from b = 0 and b = 1000 s/mm^2^ based on the conventional diffusion tensor model (Fig. 1).

### Image processing with TBSS

Voxel-wise statistical analysis of diffusion MRI data was carried out using TBSS (tract-based spatial statistics^32^), a part of FMRIB Software Library 4.1.5 (FSL, Oxford Centre for Functional MRI of the Brain, UK; www.fmrib.ox.ac.uk/fsl).^33^ All subjects’ FA data were then aligned into a common space by using the FSL nonlinear registration tool (FNIRT). Because this study involved patients with hydrocephalus, we chose to generate a study-specific target FA image. Every image was transformed into MNI152 space by combining the nonlinear transform to the target FA image with the affine transform from that target to MNI152 space. Next, the mean FA skeleton was created so that it represents the centers of all tracts common to the group. All subjects’ FA data were projected onto a mean FA skeleton. The FA threshold for skeletonization varied between 0.15 and 0.30. It was confirmed that the TBSS default threshold value of 0.20 was working well for the current data. Therefore, the subsequent analyses were performed using FA threshold of 0.20. The ADC, axial diffusivity (AD), radial diffusivity (RD), and MK maps were also projected onto the mean FA skeleton by applying the same transform as that for the FA images.

Voxel-wise statistics of the skeletonized data were analyzed with the FSL randomize tool to test for group differences between the controls and the patients with iNPH, adjusted for age and sex. We used a *t* test with 5000 permutations and statistical inference by using threshold-free cluster enhancement (TFCE), with *P* < 0.05, corrected for multiple comparisons, considered to be significant. Within the patient group, voxel-wise associations between each diffusion metric (FA, ADC, and MK) and the cognitive measure were examined by use of a nonparametric linear regression model.

### Quality assessment of the TBSS registration and MK image

As TBSS analysis for iNPH contains considerable misregistration,^16^ all the TBSS results were back-projected to each native FA map by using the “tbss_deproject” command of the FSL. All voxels were brought back from the mean FA skeleton to the subject’s native space by inverting the nonlinear registration used in TBSS process. The back-projected TBSS results were inspected for misregistration by superimposing them onto the native FA maps.

We also evaluated the quality of MK map in each subject. DKI analysis is prone to calculation error due to image noise, resulting in black or white dots in MK maps. Here voxels with MK value outside the physiologically reasonable range (0≦MK≦2) were judged as containing calculation error.^31^ Though this range of acceptable MK value was set originally presuming normal white matter, recent studies have suggested that MK comes into this range even in severely pathological tissues like glioma and multiple sclerosis.^34,35^ Using the back-projected FA skeleton as an ROI, the rates of error voxels were compared between the controls and the iNPH patients using Student’s t-test.

## Results

### Quality assessment of the TBSS registration and MK image

**Fig. 2.**
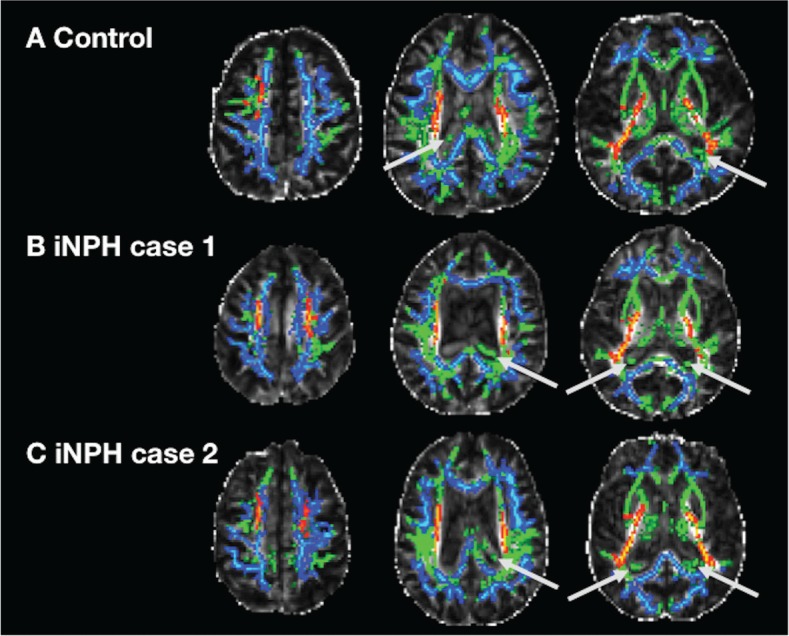
Back-projected tract-based spatial statistics (TBSS) results of representative cases, (**A**) normal control, (**B**, **C**) idiopathic normal pressure hydrocephalus (iNPH) patients. The results are overlaid on the native fractional anisotropy (FA) maps. The arrows indicate the areas where back-projected voxels from the TBSS mean FA skeleton are located in the lateral ventricles, i.e., misregistered voxels. In these examples, red color represents areas with increased FA in iNPH patients compared with the controls, while blue color represents areas with decreased FA.

Examples of back-projected TBSS results are given in Fig. 2. The back-projected TBSS results were located in the lateral ventricles for part of the corpus callosum, fornix, and the inferior part of the cingulum, indicating misregistration. Therefore, these areas were excluded from the subsequent interpretation of the TBSS results. The rate of MK calculation error within the white matter of interest (the white matter mask is shown in green in Fig. 2) did not differ significantly between the controls and the iNPH patients (1.31 ± 0.42% for the controls, 1.34 ± 0.49% for the iNPH patients).

### TBSS analysis

**Fig. 3.**
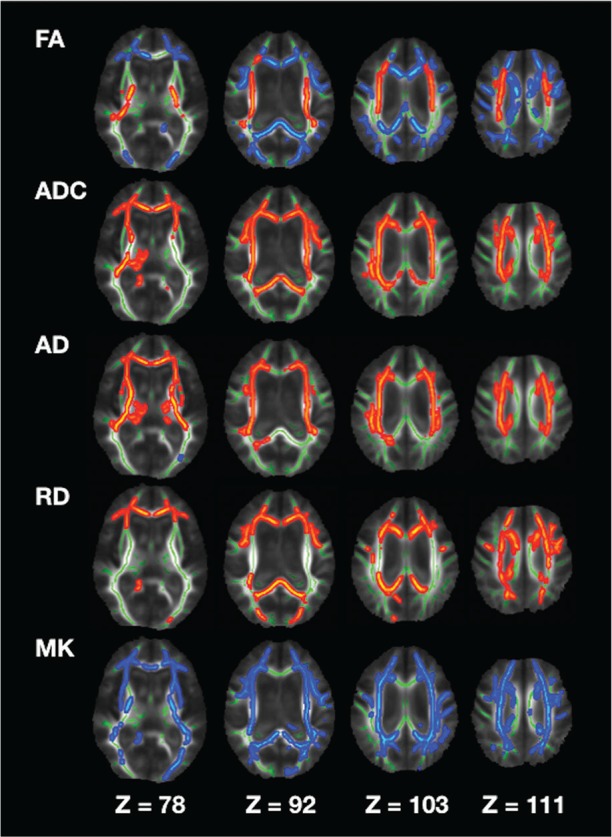
Comparison between the idiopathic normal pressure hydrocephalus (iNPH) patients and the controls. The areas with significantly increased values in the iNPH patients are marked with red-yellow, and the areas with significantly decreased values in the patients are marked with blue-light blue (*P* < 0.05, corrected for multiple comparisons). AD, axial diffusivity; ADC, apparent diffusion coefficient; FA, fractional anisotropy; MK, mean kurtosis; RD, radial diffusivity

The group comparison showed significant FA increases in the posterior limb of the internal capsule and the superior longitudinal fasciculus (SLF) in the iNPH patients compared with the controls, and significant FA decreases in the corpus callosum and the fronto-parietal subcortical white matter. Significant increases in ADC were observed in the corpus callosum, anterior and superior corona radiata, and the internal capsules. Comparison of the ADC results with those of AD and RD suggested that increase of AD predominated in the projection fiber regions like internal capsule and corona radiate, while RD increase is the main source of ADC increase in the subcortical areas. MK analyses revealed significant decreases in the SLF, inferior fronto-occipital fasciculus (IFO), anterior and superior corona radiata, posterior cingulum, internal capsules, and fronto-parietal subcortical white matter in iNPH patients (Fig. 3).

**Fig. 4.**
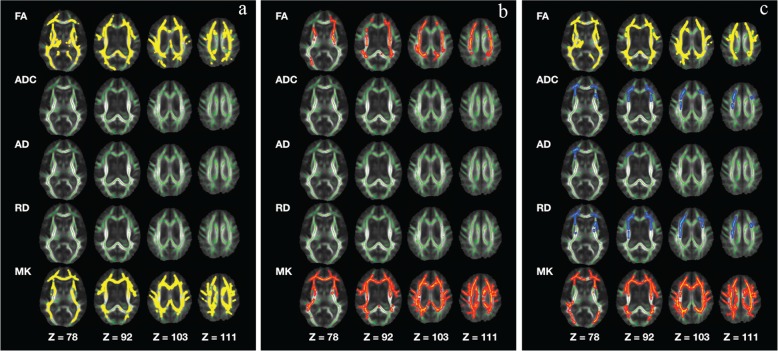
Correlation with cognitive measures in patients with idiopathic normal pressure hydrocephalus (iNPH). (**a**) Correlation with mini-mental state examination (MMSE). (**b**) Correlation with frontal assessment battery (FAB). (**c**) Correlation with trail making test A (TMT-A). Voxels with positive correlation are marked with red-yellow, while voxels with negative correlation are marked with blue-light blue (*P* < 0.05, corrected for multiple comparisons). The mean fractional anisotropy (FA) skeleton is shown in green. Because TMT-A is measured by time taken to complete the task, the design matrix is defined so that a shorter time means better cognitive function. AD, axial diffusivity; ADC, apparent diffusion coefficient; MK, mean kurtosis; RD, radial diffusivity

FA and MK showed positive correlations with cognitive function in similar regions, predominantly in the frontal lobe. Specifically, both FA and MK showed positive correlations with scores on the MMSE, FAB, and TMT-A in the SLF, IFO, inferior longitudinal fasciculus (ILF), corona radiata (anterior, superior, and posterior), the internal capsules, the corpus callosum, and the cingulum. In the fronto-parietal subcortical white matter, positive correlations were observed in more extensive areas in MK analyses than in FA, especially for FAB (Fig. 4). ADC, AD, and RD analyses showed no significant correlations with MMSE and FAB, while negative correlation with TMT-A was observed in the limited portion of the frontal deep white matter.

## Discussion

In this study, cognitive measures in iNPH demonstrated positive correlations with the FA and MK values of the white matter predominantly in the frontal lobe, suggesting the capability of these diffusion metrics as biomarkers for cognitive dysfunctions in iNPH. Specifically, voxels with significant correlation with FAB showed more confined distribution to the frontal lobes as compared to those with MMSE. FAB and TMT-A is designed focusing on assessment of frontal lobe executive function, while MMSE is a gross measure of cognitive impairment including other functional domains like temporal orientation and language.^36^ Moreover, MK analyses represented significant correlations in more extensive areas of the fronto-parietal subcortical white matter, indicating MK can play complementary roles to FA. For the comparison with the controls, we replicated findings from several previous studies^12–17^ that reported FA is increased in the CST and is decreased in the fronto-parietal subcortical white matter in iNPH.

To date, few studies have investigated associations between diffusion abnormalities and cognitive impairment in iNPH. Our results are consistent with a previous study^19^ reporting that the FAB score was correlated with the FA in the frontal and parietal subcortical white matter, and with the classic theory that cognitive impairment in iNPH is predominantly due to frontal subcortical dysfunction.^2,11^ DTI parameters of the frontal white matter have also been reported to correlate with frontal lobe executive dysfunction in patients with Parkinson disease^37^ and essential tremor.^38^ Our TBSS results demonstrated correlations with FAB and TMT-A predominantly in the frontal lobes but also extending into the temporo-parietal lobes, probably reflecting that executive functions are sensitive, but not specific, to frontal lobe functioning.^39^ Specifically, it is reported in healthy subjects that executive function is mainly correlated with diffusion metrics in the frontal white matter and in the SLF, while information processing speed, which is considered to affect FAB and TMTA scores, is correlated with those in the cingulum, corona radiate, ILF, parietal white matter, and in the thalamus.^40^

The exact pathological substrate for the MK decrease remains unclear, because MK is just a measure of deviation from the Gaussian distribution without any biophysical models. However, MK usually behaves in the same direction as FA in most of the cases, and its decrease is thought to represent tissue simplification that is likely associated with neuronal shrinkage^21,22^ and decreased axonal density.^23^ Several pathological backgrounds of cognitive deficits in iNPH have been proposed, including CSF stagnation and a decrease in the clearance of pathogenic macromolecules like tau and β-amyloid,^41^ and ischemic damages due to impairment of the periventricular arterioles and venules,^42^ which are considered as possible to cause decrease in MK.

In this study, although the wide spread ADC increase in iNPH patients suggested impaired white matter integrity, FA results showed mixture of increase and decrease probably reflecting the different distribution of AD increase and RD increase. In the projection fiber regions near the lateral ventricle, degree of AD increase was larger than RD, resulting in increase of FA, the measure of directionality. This FA increase potentially limits the ability of FA analyses to be correlated with symptoms in iNPH, at least in a way similar to other diseases, where FA is expected to decrease along with the degree of pathological changes. Meanwhile, MK analyses demonstrated significant decrease in both areas with FA increase and decrease, suggesting it can play a complementary role to FA analyses. Although the MK results from group comparison gave similar information to those of ADC, AD, and RD, MK demonstrated better correlation with the cognitive measures as compared to these metrics. Though conventional parameters like FA and ADC are apparently valuable, they have limitations that assumption of Gaussian distribution is not always true *in vivo*. On the contrary, FA and ADC are relatively robust among varying scan protocols,^43^ while MK is sensitive to scan parameters, especially SNR. In this study, the SNR of 35:1 at b = 0 s/mm^2^ will result in SNR of 4.7:1 and 2.9:1 at b = 2000 s/mm^2^ and b = 2500 s/mm^2^,^44^ respectively, which are not perfectly sufficient. However, to satisfy SNR requirement of 10:1 at b = 2500 s/mm^2^ , the SNR at b = 0 s/mm^2^ should be over 120:1,^44^ which is very challenging and almost unrealistic in clinical exam. Instead, we acquired three volumes at relatively low b values (b = 500, 1000, and 1500 s/mm^2^) where SNR can be kept sufficient, expecting it would improve DKI curve fitting by increasing data points. The present results suggest MK can play complementary roles to conventional parameters like FA and ADC in the evaluation of white matter microstructural alteration.

This study has several limitations. First, we cannot completely rule out the presence of other causes of dementia in the participants, because the iNPH patient population substantially overlaps with those at risk of vascular dementia and Alzheimer’s disease,^7^ and perfect exclusion criteria is not realistic. Second, we do not have a correlation between the diffusion metrics and treatment outcome. Third, we did not acquire data with optimized DTI protocol separately, but used part of the multi-b value data to calculate FA and ADC. This may have diminished the quality of our FA and ADC analyses, considering some discrepancy with previous studies reporting that ADC correlated with cognitive dysfunctions.^37,38,45,46^ For example, the higher the maximum b value is, the TE becomes longer, resulting in low SNR of the low-b (b = 1000 s/mm^2^) images compared to those obtained with optimized TE for b = 1000 s/mm^2^ . Finally, we are lacking a golden standard to judge which diffusion metric is the best biomarker for dementia in iNPH, although the observed differences between FA and MK results raise expectations that MK can give additional information to the conventional parameters like FA and ADC.

## Conclusion

In summary, both FA and MK correlated well with cognitive impairment in iNPH. FA and MK results showed slight but considerable differences in correlation with frontal executive functions, suggesting DKI may play a complementary role to conventional FA and ADC analyses. Further studies on correlation with treatment response are needed to establish the utility of diffusion metrics as biomarkers for dementia in iNPH.
